# Spatial Homogeneity of Bacterial Communities Associated with the Surface Mucus Layer of the Reef-Building Coral *Acropora palmata*

**DOI:** 10.1371/journal.pone.0143790

**Published:** 2015-12-14

**Authors:** Dustin W. Kemp, Adam R. Rivers, Keri M. Kemp, Erin K. Lipp, James W. Porter, John P. Wares

**Affiliations:** 1 Odum School of Ecology, University of Georgia, Athens, GA, 30602, United States of America; 2 US Department of Energy, Joint Genome Institute, Walnut Creek, CA 94598, United States of America; 3 Department of Environmental Health Science, University of Georgia, Athens, GA, 30602, United States of America; 4 Department of Genetics, University of Georgia, Athens, GA, 30602, United States of America; Pennsylvania State University, UNITED STATES

## Abstract

Coral surface mucus layer (SML) microbiota are critical components of the coral holobiont and play important roles in nutrient cycling and defense against pathogens. We sequenced 16S rRNA amplicons to examine the structure of the SML microbiome within and between colonies of the threatened Caribbean reef-building coral *Acropora palmata* in the Florida Keys. Samples were taken from three spatially distinct colony regions—uppermost (high irradiance), underside (low irradiance), and the colony base—representing microhabitats that vary in irradiance and water flow. Phylogenetic diversity (PD) values of coral SML bacteria communities were greater than surrounding seawater and lower than adjacent sediment. Bacterial diversity and community composition was consistent among the three microhabitats. Cyanobacteria, Bacteroidetes, Alphaproteobacteria, and Proteobacteria, respectively were the most abundant phyla represented in the samples. This is the first time spatial variability of the surface mucus layer of *A*. *palmata* has been studied. Homogeneity in the microbiome of *A*. *palmata* contrasts with SML heterogeneity found in other Caribbean corals. These findings suggest that, during non-stressful conditions, host regulation of SML microbiota may override diverse physiochemical influences induced by the topographical complexity of *A*. *palmata*. Documenting the spatial distribution of SML microbes is essential to understanding the functional roles these microorganisms play in coral health and adaptability to environmental perturbations.

## Introduction

Reef-building corals host diverse assemblages of internal and external microbiota (i.e., archaea, bacteria, cyanobacteria, fungi, protists, and viruses) [1,2] that collectively make up the coral holobiont [3–5]. Bacteria found within coral surface mucus layer (SML) are essential components of the coral holobiont and have crucial roles in nutrient cycling and disease prevention [4–6]. Under non-stressful conditions, beneficial microbial assemblages are often the first line of defense and are thought to out-compete and prevent infection of opportunistic pathogens. When environmental perturbations occur (e.g., coral bleaching or nutrient enrichment), these microbial assemblages are often altered and the occurrence of coral disease can increase substantially (reviewed by: [4,6,7]).

Reef-building corals are topologically complex organisms. Although skeletal growth produces stationary structures on the reef, the three-dimensional complexity of these stationary forms creates highly diverse photic environments [8–10] and fluid dynamic environments around the coral [10,11]. Given the potential for rapid turnover of the corals’ SML bacterial community and the potential importance that this community plays in metabolism and defense against water-borne pathogens, one might predict high variance in composition and abundance of these communities across such topologically complex forms.

To date, within-colony bacterial distribution has been studied for only two corals, the branching finger coral, *Porites furcata* [3] and the boulder star coral, *Orbicella annularis* [12]. Spatial analysis of the highly branched finger-coral, *P*. *furcata* revealed a significant level of within-colony heterogeneity within its SML bacterial communities. This within-colony heterogeneity of *P*. *furcata* is consistent with the hypothesis that holobiont composition responds strongly to environmental variations, which occur across a topologically complex surface. The hemispheroidal boulder coral, *O*. *annularis*, also exhibited considerable within-colony variation of bacterial assemblages between the tops and sides despite its simple geometric shape [12]. Extrapolating these results to corals with an even greater degrees of topological complexity, such as the large branching elkhorn coral, *Acropora palmata* would suggest that these highly rugose corals would have either a similar or higher degree of SML bacterial community heterogeneity.

Previous studies that have examined the *A*. *palmata* holobiont examined samples from one region [13,14] or have combined samples into a single composite for analysis [15], while disregarding the possibility of within-colony SML bacterial niche diversification. To date, there is little information to confirm these sampling assumptions, particularly among large, branching corals with inherently high spatial variability. To better understand the spatial variability and within-colony distributions of coral SML bacterial assemblages we sequenced the V1-V2 region of the 16S rRNA gene to compare potential differences from three spatially distinct regions of the threatened Caribbean reef-building coral, *A*. *palmata*. Contrary to our expectations, we found the SML associated microbial communities of *A*. *palmata* to be homogeneous.

## Material and Methods

### Coral Mucus, Seawater, and Sediment Sampling and Processing

**Fig 1:**
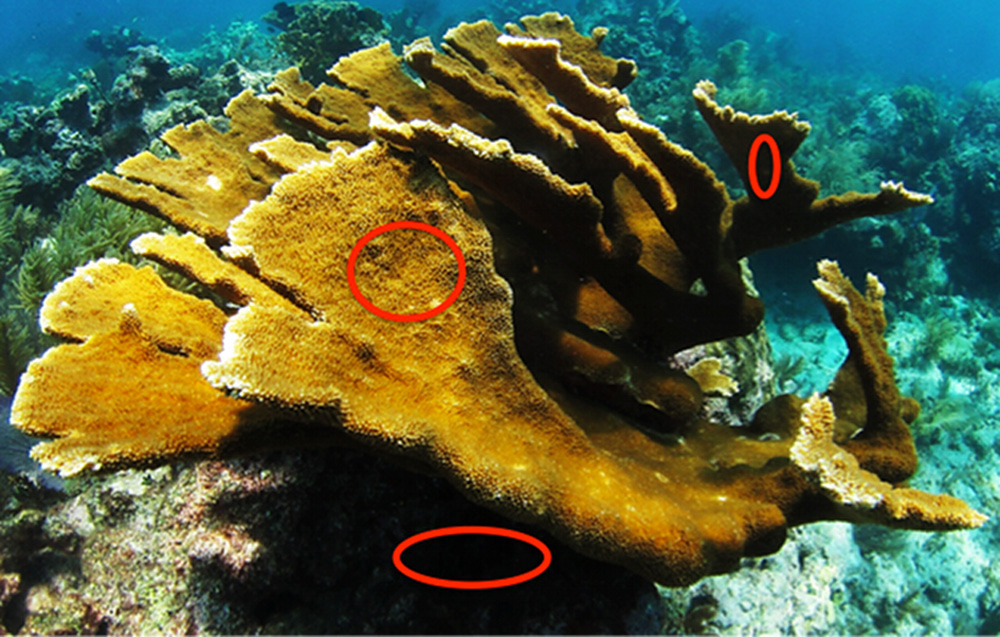
Regions of the surface mucus layer (SML) sampled (uppermost, underside, base) from *A*. *palmata*.

Colonies (n = 4) of *Acropora palmata* were sampled from Looe Key (3 m depth; N 24°32.724’ W81°24.360’), located in the Florida Keys National Marine Sanctuary during June 2011 using SCUBA. Sterile, needleless syringes (10 ml) were used to gently withdraw the surface mucus layer (SML) of *A*. *palmata* from biologically relevant regions: uppermost (high irradiance), underside (low irradiance), and the base of replicate coral colonies (Fig 1). All samples were collected from colonies that visually appeared healthy (i.e. no bleaching or disease lesions). Seawater was collected, in 10-ml syringes, approximately 1 m directly above sampled corals. Sediment was scooped into 50-ml conical vials directly from the base of sampled corals. All samples were placed on ice, transported to the laboratory, and processed within 2 h of collection. Syringe contents were transferred into 15 ml conical tubes, vortexed for 5–10 s, and 2 ml were pelleted by centrifuging at 20,000 x *g* for 20 min. Two-ml aliquots from sediment samples were processed in the same manner. After centrifugation, supernatant fluids were carefully poured off and pellets were frozen at -20°C.

### DNA extraction and 16S rDNA amplification

DNA was extracted following the protocol described in Boström et al. [16]. Briefly, pellets were thawed, dissolved in 175 μl of lysis buffer (400 mM NaCl, 750 mM sucrose, 20 mM ethylenediaminetetraacetic acid (EDTA), 50 mM Tris-HCL pH 9.0), followed by a lysozyme digestion (1 mg ml^-1^ final) for 30 min at 37°C and a proteinase K (100 μg ml^-1^ final) / sodium dodecyl sulfate (1% *v*/*ν* final) overnight digestion at 55°C. DNA was recovered with 50 μg Baker’s yeast tRNA as a coprecipitant [17] with 1:10 vol 3M NaOAc, pH 5.2, and 0.6 volume of isopropanol and incubated at -20°C for 1 h. Samples were placed in a microcentrifuge at 4°C and spun for 20 min (20,000 x *g*). Supernatant fluids were discarded and pellets were washed with 500 μL of 70% ethanol and then spun a second time (4°C, 20 min, 20,000 x *g*). DNA pellets were dried in a SpeedVac (Thermo Scientific, Asheville, NC) for 20 min and dissolved in 20 μl of 10 mM Tris-HCl pH 7–8. Extracted DNA was stored at -20°C until use.

The V1-V2 region of the 16S ribosomal rRNA gene was amplified using error-correcting DNA barcoded PCR primers developed by Hamady et al. [18] for multiplexed pyrosequencing. The forward primer (5’-GCC TTG CCA GCC CGC TCA GTC AGA GTT TGA TCC TGG CTC AG-3’) contained the 454 Life Sciences primer B sequence, the broadly conserved bacterial primer 27F, and a two-base linker sequence (“TC”). The reverse primer (5’–GCC TCC CTC GCG CCA TCA GNN NNN NNN NCA TGC TGC CTC CCG TAG GAG T- 3’) contained the 454 Life Sciences primer A sequence, a unique 8-nt error-correcting Golay barcode, (designated by NNNNNNNN), the bacterial primer 338R, and “CA” linker sequence [18]. Four replicate 50-μl PCR amplifications were prepared for each sample and amplified products were combined. Each PCR consisted of 26 μl of Promega ultra pure water (Madison, WI, USA), MgCl_2_ (2.5 mM), dNTPs (final concentration 200 μM), 1u of AmpliTaq Gold DNA polymerase LD (Invitrogen, Carlsbad, CA, USA) and 2 μl of genomic DNA (~ 5 ng). Negative controls, Promega ultra pure water, were run in parallel to all environmental samples to check for potential primer or sample DNA contamination. Triplicate reactions were held at 95°C for 5 min to denature DNA, then 30 cycles of 94°C for 30s, 55°C for 60s, and 72°C for 90s, and a final extension at 72°C for 10 min. All PCR products were loaded on 2% agarose gels and electrophoresed for 1h at 60V to verify amplification and screen for potential contamination.

### Pyrosequencing sample preparation and analysis

The composite samples were purified using StrataPrep PCR Purification Kits (Agilent Technologies, Santa Clara, CA, USA). Coral and sediment samples were submitted to the Georgia Genomics Facility (Athens, GA, USA) and seawater samples were submitted to Molecular Research Laboratories (Shallowater, TX, USA) for library preparation, emulsion PCR, and sequencing on a 454 Life Sciences Genome Sequencer FLX (Roche, Branford, Connecticut, USA).

Sequences from the V1-V2 16S rRNA region were analyzed using a suite of tools available in QIIME version 1.8 [19]. Sequences libraries were demultiplexed and processed through Denoiser [20] to remove homopolymer errors characteristic of pyrosequencing. The initial assignment of operational taxonomic units at the 97% similarity level (OTUs_0.03_) was made using the *denovo* OTU picker script in QIIME with Usearch for clustering [21]. OTUs_0.03_ with 2 or fewer reads were removed (5% of all sequences) to reduce the artificial inflation of diversity estimates due to sequencing errors. Reads were aligned with PyNAST [22] and Chimera Slayer [23] was used to identify chimeric reads. Source tracker [24] was used to identify samples that contained either significant laboratory or seawater contamination. Only one sample, taken from the SML at the base of colony Ap62, was determined to be 50% PCR contamination and was omitted from further analyses. No other samples showed contamination.

Taxonomic assignment of OTUs was completed using the Ribosomal Database Project (RDP) classifier and the RDP dataset [25,26] at a confidence threshold of 70%. OTUs_0.03_ classified as chloroplast or mitochondria by RDP were removed and BLAST [27] searches were conducted to verify that mitochondria and chloroplast sequences from *Acropora* sp. and *Symbiodinium* sp. were not present in the final libraries. Approximate maximum-likelihood phylogenetic trees were built using FastTree2 [28] and samples were ordered by their UniFrac dissimilarity matrix [29] using the complete linkage hierarchical clustering method in R [30]. Taxa were represented at the phylum, class or family level depending on their abundance in the samples and plotted using rColorBrewer color palates in R [30].

To examine potential differences in alpha-diversity, samples were rarefied to 10 sequencing depths 10 times and observed OTUs_0.03_, whole tree phylogenetic distance (PD), Chao1, and Shannon-Wiener diversity were calculated using the alpha diversity script in QIIME (S1 Table). Beta diversity, the differentiation between the microbial communities, was estimated by comparing weighted UniFrac indices to evaluate the relative abundances as well as presence of OTUs_0.03_ [31]. To visualize the taxonomic variation between samples and sample sources, these distance matrices were then used in a principal coordinate analysis (PCoA) using the QIIME scripts principal_coordinates.py. PCA data were plotted with R. The significance of differences in community structure between sample types (coral, seawater, sediment) and among coral location (uppermost, underside, base) was evaluated by Permutational ANOVA of a Bray-Curtis dissimilarity matrix based on phylogenetic distance using the Adonis method in the R package Vegan [32]. Scripts to fully reproduce the analyses in this paper are available at https://github.com/arivers/CoralPaper2015.

## Results

**Fig 2:**
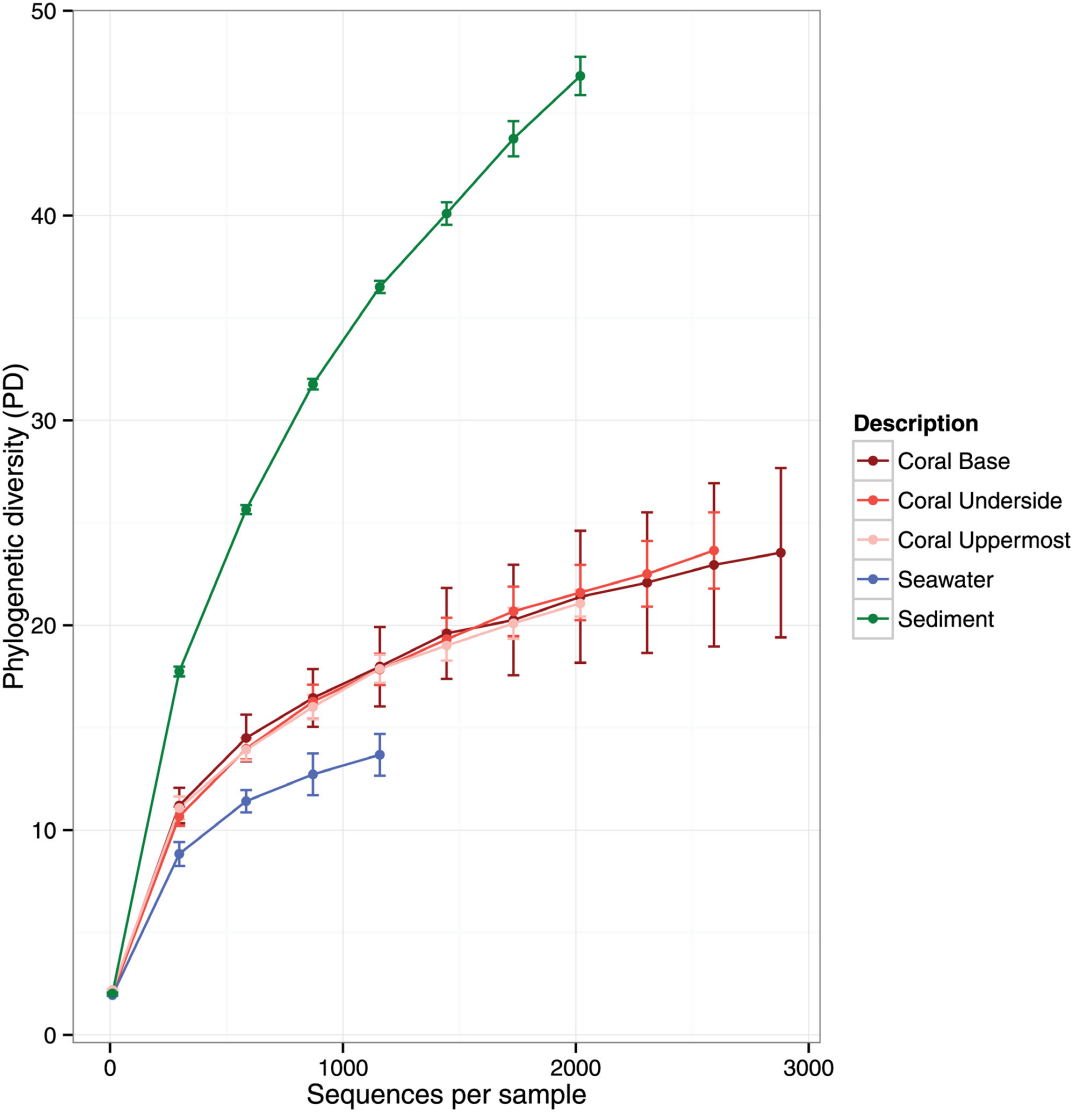
Measurements of biodiversity within each coral region, surrounding reef seawater, and sediment sampled. Rarefaction curves for microbial communities calculated for 3 coral surface types (n = 3–4; 11 samples), reef seawater (n = 4), and sediment samples (n = 2). Operational taxonomic unit (OTU_0.03_) richness rarefaction curves were estimated by phylogenetic diversity (PD).

We obtained 64,022 high-quality 16S rRNA gene pyrosequencing reads (mean corrected length 313 nt; BioProject database ID PRJNA230817) from three regions located on *Acropora palmata* colonies (uppermost, underside, and base), adjacent sediments, and seawater 1 m above coral colonies (Fig 1). To investigate phylogenetic richness within each sample, rarefaction curves for phylogenetic diversity (PD) were calculated for each sample type. Using the highest sampled number of sequences available in all samples (1158) as a comparison point (Fig 2, Table 1), microbial PD was greatest in the sediment (36.52 ± 0.30 SE) and lowest in the water samples (13.68 ± 1.02). All three coral regions examined had similar PD values (uppermost, 17.88 ± 0.69 SE; underside, 17.84 ± 0.77 SE; base, 17.98 ± 1.94 SE).

**Table 1:** Diversity by sample type at the rarefaction depth of 1158 sequences averaged for 10 random sequence subsets. Numbers within brackets indicate standard errors.

Sample Type	Sample Number	Observed OTUs_0.03_	Chao1	Shannon-Wiener	Phylogenetic Diversity
Coral Uppermost	4	182.60 (6.74)	305.86 (7.30)	6.14 (0.12)	17.88 (0.69)
Coral Underside	4	182.58 (7.93)	321.77 (38.09)	6.05 (0.10)	17.84 (0.77)
Coral Base	3	182.20 (28.26)	309.23 (94.14)	6.01 (0.37)	17.98 (1.94)
Seawater	4	151.95 (10.91)	200.37 (22.55)	5.49 (0.15)	13.68 (1.02)
Sediment	2	436.50 (17.30)	1165.91 (129.86)	7.25 (0.09)	36.52 (0.30)

**Fig 3:**
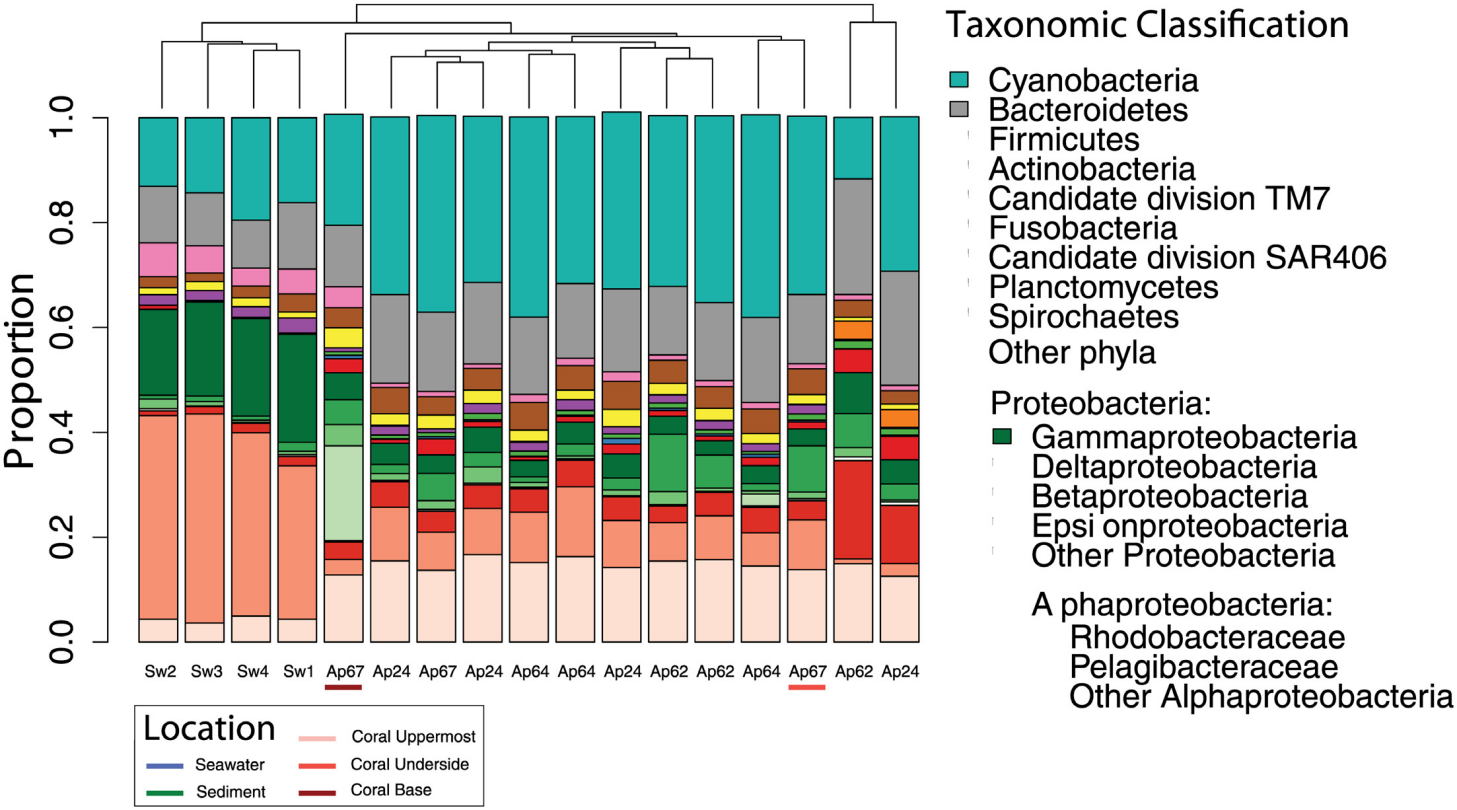
Microbial diversity in different regions of the SML of *A*. *palmata* (n = 3–4; 11 samples), reef seawater (n = 4), and adjacent sediment (n = 2).

The three sampled regions of the surface mucus layer microbiota from *A*. *palmata* colonies (base, underside, and uppermost) were dominated by similar taxa. Sequences from Cyanobacteria, Bacteroidetes, Deltaproteobacteria and Alphaproteobacteria in the orders Pelagibacterales, Rhodobacterales were the most abundant taxa, respectively (Fig 3). The two sediment samples were found to have the highest alpha-diversity (Fig 2, Table 1) with an average Chao1 diversity index 1165.91 ± 129.86 SE at a rarefaction depth of 1158 sequences. Sediment samples were dominated by sequences belonging to Bacteroidetes, Cyanobacteria, Rhodobacterales, and Gammaproteobacteria, respectively. Sediment samples had a higher prevalence of Rhodobacterales than coral or seawater samples (Fig 3). Seawater samples were found to have the lowest alpha-diversity with an average Chao1 index of 200.37 ± 22.55 SE at a rarefaction depth of 1158 sequences (Table 1). Seawater samples had a high abundance of Pelagibacteriaceae, Gammaproteobacteria, Cyanobacteria, Bacteriodetes, and Firmicutes (Fig 3).

**Fig 4:**
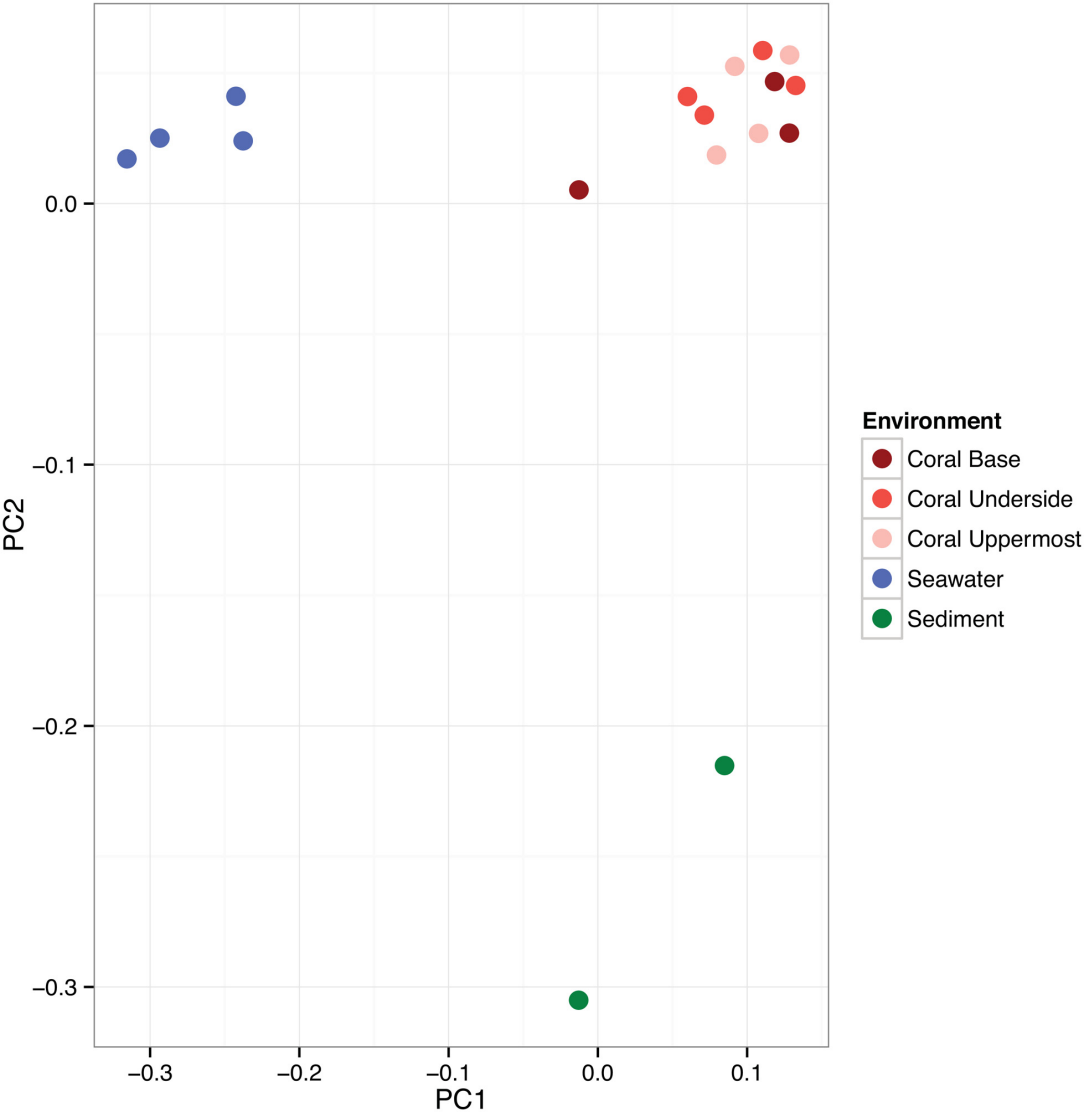
Principal coordinate analysis of beta-diversity metrics among microbial samples from *A*. *palmata*. Samples are coded by source tissue or environment (base, underside, uppermost, reef seawater, and sediment).

Community composition was significantly different between coral, seawater, and sediment. No significant differences in community assemblages were found between any of the three coral regions or between any of the individual coral colonies (Fig 3). The PCoA results clearly separate sediment, seawater and coral samples, with the associated first principle coordinate explaining 53% of the taxonomic variation and sediment sample variation further separated by the second principle coordinate axis, explaining another 20% of the taxonomic variation (Fig 4). Permutational ANOVA analyses based on a Bray-Curtis dissimilarity matrix of phylogenetic distance (S2 Table) revealed that coral associated microbial communities were structurally different from those in seawater (R2 = 0.82, p = 0.0008) and those in sediments (R2 = 0.58, p = 0.013). There was no detectable community-level difference among the microbiota of the uppermost, underside, and base of *A*. *palmata* (R2 = 0.20, p = 0.51), indicating homogeneity among these coral regions.

## Discussion

Vertical stratification of bacterioplankton is commonly found in marine environments [33–35]. Similarly, physicochemical factors (e.g., temperature, pH, and nutrients) have been shown to influence prevalence and diversity of surface-attached bacteria [36]. The three-dimensional structure of *A*. *palmata* colonies creates distinct microenvironments, separated by only a few centimeters, which differ in levels of irradiance and water velocity [10]. Microbes that tolerate high irradiance and ultraviolet radiation would be expected to have disproportionate abundances in the uppermost (exposed) regions of the coral [37]. Surprisingly, in this study, coral-associated microbiota from the surface mucus layer (SML) of *A*. *palmata* were found to have similar assemblages between uppermost (high irradiance), underside (low irradiance), and the base of replicate coral colonies (Figs 3 and 4).

This study documents the spatially homogeneous nature of SML bacterial communities associated with *A*. *palmata* during non-stressful conditions and offers helpful guidelines for study design. In 2006, *A*. *palmata* was classified as critically threated under the U.S. Endangered Species Act, necessitating the development and application of benign sampling techniques. Our data shows clear separation of microbial communities associated with seawater versus the *A*. *palmata* SML (Fig 4; Permutational ANOVA, R2 = 0.82, p = 0.0008), providing support for the use of non-destructive, syringe-sampling methods. Moreover, our data validates sampling schemes that collect material from a single location on an *A*. *palmata* colony.

The baseline diversity estimate provided here can be used to determine the sequencing effort needed to characterize microbiota found in the SML of *A*. *palmata*. We found a similar level of alpha diversity, or species richness, in SML microbial communities of *A*. *palmata* in the Florida Keys (~180 observed OTUs_0.03_ and Shannon-Wiener diversity index ~6, Table 1) compared to a previous investigation utilizing pyrosequencing to examine *A*. *palmata* SML microbial communities in the Mexican Caribbean (~70–100 observed OTUs_0.03_ and Shannon-Wiener diversity index ~3) [15]. However, comparisons across 16S rRNA amplicon studies are often confounded by differences in sequencing depth and bioinformatic processing. Sunagawa and colleagues [14] report higher values of observed and Chao1 predicted OTUs_0.03_ for microbiomes of *A*. *palmata* in Panamá. These differences may be attributable to their (1) sampling of coral fragment homogenates, which includes microbes present in coral tissue and skeleton in addition to mucus, (2) inclusion of rare OTUs_0.03_ (with 2 or fewer reads) in analyses of alpha-diversity, and (3) use of a different variable region of the 16S rRNA gene.

This is the first fine-scale analysis of spatial variability within SML microbial communities of *A*. *palmata*. Previous work investigated within-colony spatial variability of the Caribbean reef-building corals *Porites furcata* and *Orbicella annularis* and found microbial assemblages to exhibit patterns of within-colony heterogeneity [12,38]. These contrasting results—within-colony heterogeneity in *P*. *furcata* and *O*. *annularis* and homogeneity in *A*. *palmata*—may be explained, in part, by whether or not the coral simultaneously hosts diverse assemblages of photosymbionts. Microbial communities of marine invertebrates, including corals, have been shown to be influenced by the presence of photosymbionts [39]. Endosymbiotic dinoflagellates (*Symbiodinium* spp.) provide the majority of metabolic carbon to most tropical and sub-tropical reef-building corals [40]. *Symbiodinium*-derived products include carbohydrates, fatty acids, sugars, starches, and amino acids (Reviewed by: [41,42]), and likely influence the composition of coral mucus. *Acropora palmata* colonies in the Florida Keys are typically dominated by a single strain of *Symbiodinium* [43]. By contrast, species of the *O*. *annularis* complex are known to commonly associate simultaneously with diverse assemblages of *Symbiodinium* [44–46]. These contrasting results suggest that within-colony spatial heterogeneity in dominant *Symbiodinium* types may beget greater spatial heterogeneity in SML microbial communities. However, few studies have explicitly addressed this question [47,48] and fine scale analyses of how spatial heterogeneity of dominant *Symbiodinium* influences mucus composition are needed [39,49].

The homogeneity patterns described in this study also suggest that *A*. *palmata* strongly regulates its SML microbiota during non-stressful conditions. Multiple mechanisms for host regulation of microbiota associated with corals have been suggested and include the ability of coral to 1) detect specific micro-organism-associated molecular patterns (MAMPs) and regulate components of the innate immune system, 2) excrete compounds with antimicrobial properties to suppress the growth of undesirable microbiota, and/or 3) release compounds that attract and maintain keystone taxa capable of stabilizing the community and preventing opportunistic invasions from undesirable microbes (reviewed by: [2]). Full genome analysis of the Pacific coral *Acropora digitifera* revealed an extraordinarily high number of loci encoding for domains within MAMP recognition receptors compared to a representative range of metazoans for which whole-genome data are available [50]. More investigations of host-regulatory mechanisms in symbiotic corals are needed to understand whether the extraordinary diversity of MAMP recognition receptors—representing the potential for recognition and strong regulatory control over microbial symbionts—uncovered in *Acropora* is unique to this genus, or comparable to other coral species.

Coral-associated microbiota found on the SML are highly diverse and play vital roles in coral health [4,51,52]. This study documents the spatially homogeneous nature and provides an important baseline diversity estimate of SML bacterial communities associated with *A*. *palmata* during non-stressful conditions. Stability of *A*. *palmata* microbial community structure may result from 1) regulatory control of the coral host 2) highly stable symbiosis with only one type of algal symbiont 3) other unknown mechanisms. The highly specific nature of the *A*. *palmata* mucus microbial community will help us define the roles these microorganisms play in assuring coral health, guarding against coral disease, and responding to environmental perturbations.

## Supporting Information

S1 Table Rarefaction results. (PDF)

S2 Table Results of permutational ANOVA based on Bray-Curtis dissimilarities of phylogenetic distance. (PDF)
