# Correction: Spatial Homogeneity of Bacterial Communities Associated with the Surface Mucus Layer of the Reef-Building Coral *Acropora palmata*

**DOI:** 10.1371/journal.pone.0146987

**Published:** 2016-01-08

**Authors:** The PLOS ONE Staff

There are errors in the color legend for Fig 3. The publisher apologizes for the error. Please see the corrected Fig 3 here.

**Fig 3:**
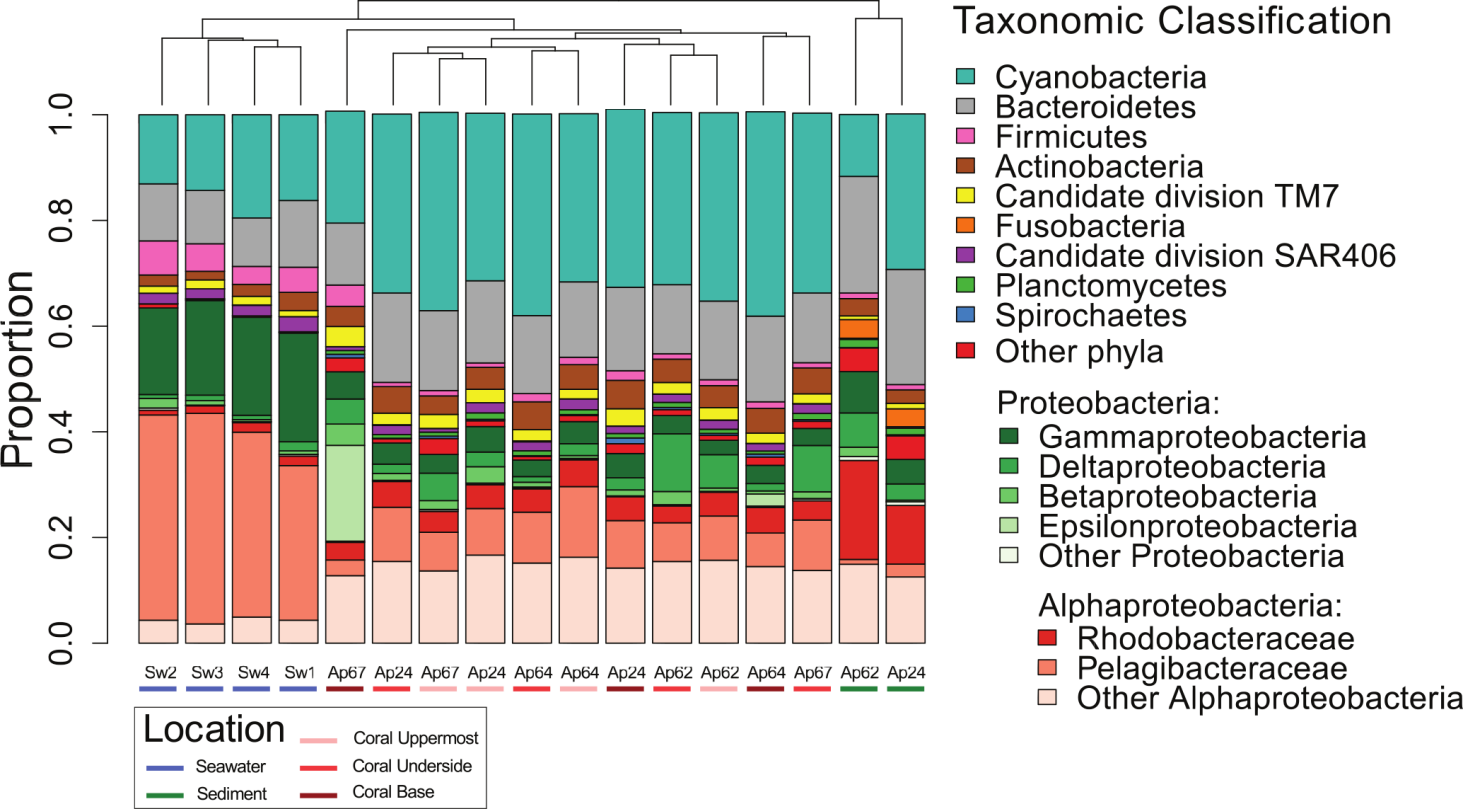
Microbial diversity in different regions of the SML of *A*. *palmata* (n = 3–4; 11 samples), reef seawater (n = 4), and adjacent sediment (n = 2).
