# Supplementary material for: Spatial Homogeneity of Bacterial Communities Associated with the Surface Mucus Layer of the Reef-Building Coral Acropora palmata
Source: PLoS One. 2015 Dec 14;10(12):e0143790. doi: 10.1371/journal.pone.0143790 (PMC4682823; doi:10.1371/journal.pone.0143790)
Supplement: S1 Table — (PDF) [file pone.0143790.s001.pdf]

S1 Table. Rarefaction results. Samples were rarefied to ten different depths ranging from the median sample size (2880) to 10 sequences. Average and standard error of phylogenetic distance (PD), observed $OTUs_{0.03}$, Chao1, and Shannon-Wiener diversity indices were calculated based on 10 random sequence subsets of each sample.

Sample type	Rarefied Seqs/Sample	PD Ave.	PD Err.	Chao1 Ave.	Chao1 Err.	Observed OTUs Ave.	Observed OTUs Err.	Shannon Ave.	Shannon Err.
Coral Base	10	2.114	0.116	30.544	7.488	8.833	0.419	3.074	0.091
Coral Base	297	11.203	0.868	174.978	37.05	92.833	10.489	5.657	0.336
Coral Base	584	14.498	1.138	243.79	53.417	135.067	16.539	5.888	0.367
Coral Base	871	16.45	1.411	269.679	71.277	162.567	21.576	5.981	0.33
Coral Base	1158	17.981	1.936	309.226	94.144	182.2	28.263	6.008	0.374
Coral Base	1445	19.602	2.218	327.197	103.253	203.833	34.815	6.059	0.369
Coral Base	1732	20.254	2.699	352.858	121.561	216.467	41.832	6.069	0.374
Coral Base	2019	21.389	3.222	368.1	127.139	231.5	47.963	6.102	0.37
Coral Base	2306	22.08	3.429	383.807	139.148	243.433	53.7	6.112	0.383
Coral Base	2593	22.946	3.989	403.899	152.227	256.733	60.797	6.123	0.388
Coral Base	2880	23.544	4.135	414.053	159.365	266.467	65.087	6.133	0.385
Coral Underside	10	2.038	0.133	32.375	7.083	8.975	0.286	3.102	0.061
Coral Underside	297	10.672	0.461	192.813	13.668	93.35	2.348	5.711	0.08
Coral Underside	584	13.968	0.517	238.652	14.829	131.675	3.819	5.921	0.107
Coral Underside	871	16.268	0.831	300.752	34.693	159.975	3.909	6.002	0.1
Coral Underside	1158	17.842	0.766	321.767	38.094	182.575	7.929	6.051	0.103
Coral Underside	1445	19.314	1.047	345.1	36.576	203.575	8.469	6.09	0.091
Coral Underside	1732	20.678	1.209	373.072	61.216	220.15	12.086	6.12	0.101
Coral Underside	2019	21.595	1.352	386.729	50.031	233.475	11.601	6.127	0.099
Coral Underside	2306	22.507	1.604	403.909	67.095	248.8	15.274	6.148	0.105
Coral Underside	2593	23.649	1.857	424.319	72.638	262.925	18.303	6.162	0.096
Coral Underside	2880	-	-	-	-	-	-	-	-
Coral Uppermost	10	2.2	0.033	31.325	2.625	9	0.122	3.118	0.023
Coral Uppermost	297	11.076	0.563	172.063	9.973	94.525	4.361	5.827	0.106
Coral Uppermost	584	13.92	0.515	228.687	13.902	130.025	4.22	5.982	0.103
Coral Uppermost	871	16.011	0.599	274.758	8.174	161.225	4.105	6.102	0.093
Coral Uppermost	1158	17.875	0.685	305.864	7.296	182.6	6.738	6.143	0.116
Coral Uppermost	1445	19.009	0.742	325.867	27.04	202.35	5.628	6.195	0.105
Coral Uppermost	1732	20.092	0.752	337.426	15.371	218.2	5.881	6.212	0.111
Coral Uppermost	2019	21.07	0.646	348.3	14.844	232.175	5.559	6.234	0.108
Coral Uppermost	2306	-	-	-	-	-	-	-	-
Coral Uppermost	2593	-	-	-	-	-	-	-	-
Coral Uppermost	2880	-	-	-	-	-	-	-	-
Seawater	10	1.949	0.053	23.613	4.755	7.95	0.377	2.843	0.098
Seawater	297	8.837	0.584	150.406	19.687	84.65	4.908	5.218	0.145
Seawater	584	11.412	0.542	185.742	17.343	119.25	6.913	5.408	0.138
Seawater	871	12.72	1.022	196.448	21.012	139.85	11.511	5.482	0.142
Seawater	1158	13.679	1.02	200.367	22.545	151.95	10.909	5.492	0.151
Seawater	1445	-	-	-	-	-	-	-	-
Seawater	1732	-	-	-	-	-	-	-	-
Seawater	2019	-	-	-	-	-	-	-	-
Seawater	2306	-	-	-	-	-	-	-	-
Seawater	2593	-	-	-	-	-	-	-	-
Seawater	2880	-	-	-	-	-	-	-	-
Sediment	10	2.028	0.039	31.55	5.45	8.6	0.6	2.985	0.162
Sediment	297	17.747	0.237	620.281	134.483	160.5	6.3	6.435	0.117
Sediment	584	25.644	0.224	838.19	93.926	267.1	8.1	6.903	0.072
Sediment	871	31.769	0.256	1040.517	60.826	359.15	12.75	7.136	0.078
Sediment	1158	36.518	0.299	1165.908	129.863	436.5	17.3	7.249	0.089
Sediment	1445	40.099	0.549	1323.852	166.743	504.75	24.15	7.353	0.093
Sediment	1732	43.754	0.855	1480.948	192.159	569.2	32.3	7.414	0.108
Sediment	2019	46.814	0.933	1671.068	262.524	632.55	36.15	7.48	0.098
Sediment	2306	-	-	-	-	-	-	-	-
Sediment	2593	-	-	-	-	-	-	-	-
Sediment	2880	-	-	-	-	-	-	-	-
