# Supplementary material for: Spatial Homogeneity of Bacterial Communities Associated with the Surface Mucus Layer of the Reef-Building Coral Acropora palmata
Source: PLoS One. 2015 Dec 14;10(12):e0143790. doi: 10.1371/journal.pone.0143790 (PMC4682823; doi:10.1371/journal.pone.0143790)
Supplement: S2 Table — (PDF) [file pone.0143790.s002.pdf]

S2 Table. Results of permutational ANOVA based on Bray-Curtis dissimilarities of phylogenetic distance.

	**Df**	**SumsOfSqs**	**MeanSqs**	**F.Model**	**R2**	**Pr(>F)**
uppermost:underside:base	2	0.055	0.027	0.97	0.2	0.51
coral:seawater	1	2.36	2.39	60.33	0.82	0.0008
coral:sediment	1	0.59	0.59	15.19	0.58	0.013
sediment:water	1	0.16	0.16	11.7	0.75	0.067
